# Berberine protects diabetic nephropathy by suppressing epithelial-to-mesenchymal transition involving the inactivation of the NLRP3 inflammasome

**DOI:** 10.1080/0886022X.2022.2079525

**Published:** 2022-05-26

**Authors:** Zejun Ma, Lili Zhu, Shangshang Wang, Xin Guo, Bei Sun, Qilong Wang, Liming Chen

**Affiliations:** a NHC Key Laboratory of Hormones and Development, Tianjin Key Laboratory of Metabolic Diseases, Chu Hsien-I Memorial Hospital & Tianjin Institute of Endocrinology, Tianjin Medical University, Tianjin; b Tianjin Medical Devices Quality Supervision and Testing Center, Tianjin, China; c Institute of Traditional Chinese Medicine, Tianjin University of Traditional Chinese Medicine, Tianjin, China

**Keywords:** Diabetic kidney disease, renal tubulointerstitial fibrosis, epithelial-to-mesenchymal transition, NLRP3 inflammasome, berberine

## Abstract

Accumulating evidence has implicated that berberine (BBR) has a beneficial effect on diabetic kidney disease (DKD), but its mechanism is not clear. The aim of this study was to assess whether berberine could alleviate tubulointerstitial fibrosis and attenuate epithelial-to-mesenchymal transition (EMT) and its possible molecular mechanism. High-fat diet (HFD) followed by injection of STZ was used to induce diabetic rats *in vivo*. After the onset of diabetes, rats were treated with either BBR or saline for 12 weeks. *In vitro*, the human renal proximal tubular epithelial cell line (HK-2) was exposed to high glucose, with or without BBR. The influence of berberine on renal tubulointerstitial histological changes, markers of epithelial-to-mesenchymal transition (EMT) and (NOD-like receptor pyrin domain-containing protein 3) NLRP3 inflammasome expression were examined. Results showed that *in vivo*, BBR could significantly ameliorate microalbumin and renal pathologic changes in diabetic rats. Immunofluorescence showed that BBR could inhibit EMT. Furthermore, BBR could down-regulate the level of the NLRP3 inflammasome in diabetic rats. Consistently, *in vitro*, BBR suppressed high glucose-induced EMT and activation of NLRP3 inflammasome in HK-2. Our study demonstrated that BBR could inhibit high glucose-induced EMT and renal interstitial fibrosis by suppressing the NLRP3 inflammasome. BBR might be used as a novel drug to ameliorate tubulointerstitial fibrosis in DKD.

## 1. Introduction

Diabetic kidney disease (DKD) is a common and serious microvascular complication of diabetes mellitus. DKD has become the main cause of the end-stage renal disease (ESRD) [1,2]. Although DKD patients are clinically received lowering blood glucose or blood pressure treatment s, the progression of DKD is not completely prevented and finally develops to ESRD [3–5].

DKD is characterized by persistent albuminuria caused by renal interstitial fibrosis, which is triggered by the accumulation of extracellular matrix (ECM) in glomerular mesangium and tubulointerstitium and renal tubular epithelial-mesenchymal transition (EMT) [6,7]. Loss of E-cadherin and increased expression of α-smooth muscle actin (α-SMA) are the characteristics of EMT [8–10]. Control of EMT has been identified and may be used in the intervention of tubulointerstitial fibrosis in future [11]. Growing studies indicate that inflammation may be involved in the progress of tubulointerstitial fibrosis [12–14]. In addition, our previous study has shown that activation of NF-κB plays a role in renal inflammation and tubulointerstitial fibrosis in the progression of DKD [15]. Recent studies have demonstrated that the inflammasome of nucleotide binding oligomeric domain like receptor protein 3 (NLRP3) participated in the process of renal inflammation, leading to the occurrence and development of DKD [16–19]. Therefore, targeting NLRP3 inflammasome may be a positive and potential therapy for DKD.

Berberine (BBR: [C20H18NO4]^+^), an isoquinoline alkaloid extracted from Chinese herbal medicine, is one of the main components of Coptis chinensis. Studies have shown that BBR has various metabolic benefits including antioxidant, anti-inflammatory and anti-fibrosis effects. In addition, increasing evidence show that BBR can effectively improve renal injury, inhibit mesangial cell proliferation and ameliorate tubulointerstitial fibrosis, suggesting that BBR might be used as a potential treatment for DKD [20,21]. However, the renal protective mechanism of BBR on DKD remains to be further explored. This study aims to assess whether berberine alleviates renal tubulointerstitial fibrosis and alleviates EMT and its potential molecular mechanism.

## 2. Materials and methods

### 2.1. Cell

HK-2 cells were cultured in Dulbecco’s modified Eagle’s medium/F12 (DMEM/F12) medium containing 10% fetal bovine serum (FBS, Thermo Fisher Scientific) and maintained at 37 °C in a humidified atmosphere of 5% CO_2_. Cultured cells were divided into six groups: normal control group (NG group): 5.6 mmol/L glucose; osmotic pressure control group (OP group): 5.6 mmol/L glucose + 24.4 mmol/L mannitol; high glucose group (HG group): 30 mmol/L glucose; different concentrations of BBR (1, 10 or 100 μM) + high glucose group (BBR + HG group). After 48 h of BBR and HG treatment, the cells were collected for subsequent experiments.

### 2.2. Animal model and experimental protocol

T2DM was induced in rats with a high-fat diet (HFD) followed by a single tail intravenous injection of STZ as previously reported [22]. Thirty healthy 6-week-old male Sprague-Dawley (SD) rats were purchased from Beijing Huafukang Bioscience Co., Inc and housed under a 12:12 h light-dark cycle with free access to water and food. The rats were randomly divided into the normal control group (NC group, *n* = 10) and the diabetes group (*n* = 20). The normal control group was fed a standard diet continuously. The diabetes group was fed with a high-fat diet freely to induce dyslipidemia for 6 weeks, then 30 mg/kg STZ which was dissolved in 0.1 M citrate-phosphate buffer (pH 4.5) was injected intravenously. Fasting blood glucose (FBG) ≥ 16.7 mmol/L was considered a successful diabetic model. Then the diabetic rats were divided into two subgroups: the BBR group (*n* = 10) and the DM group (*n* = 10). BBR group were orally treated with BBR (150 mg/kg·d) dissolved in 0.5% carboxymethyl cellulose every day for 12 weeks. The DM group and NC group were orally given with the same volume of 0.5% carboxymethyl cellulose. Body weight and FBG were measured once per two weeks. After 12 weeks of treatment, 24-h urine, blood, and kidney samples were collected for subsequent experiments. All experimental procedures and protocols were approved by the Animal Research Committee of Tianjin Medical College.

### 2.3. Metabolic analyses

The kidney weight was measured and the ratio of kidney weight to body weight (KW/BW) was calculated. Urinary albumin excretion was evaluated as the ratio of urinary albumin to creatinine ratio (ACR). Urea nitrogen (BUN) was measured using Urea Nitrogen Colorimetric Detection Kit (Arbor Assays, Ann Arbor, MI, USA). Serum and urinary creatinine were determined by Creatinine Colorimetric/Fluorometric Assay Kit (Biovision, Milpitas, CA, USA). Serum HbA1c was determined by Glycated Hemoglobin (HbA_1_C) ELISA Kit (Wuxi DonglinSci&Tech Development Co., Ltd., Wuxi, China). Total cholesterol (TC), low density lipoprotein (LDL-C), high density lipoprotein (HDL-C), and triglyceride (TG) were determined by gel filtration high-performance liquid chromatography.

### 2.4. Histological examination

Paraffin-embedded kidney sections were cut into 4 μm thick and deparaffinized with xylene. Hematoxylin and eosin (H&E) staining were used to evaluate the renal injury. Tissue damage was examined in a blinded manner and scored according to the percentage of damaged tubules: 0, no damage; 1, less than 25% damage; 2, 25%–50% damage; 3, 50%–75% damage; and 4, more than 75% damage as reported. Paraffin-embedded kidney tissue sections were deparaffinized and stained with Masson trichrome. The extent of renal fibrosis was assessed based on the amount of collagen deposition using an inverted microscope (DMi8, Leica, Germany), and collagen quantification was analyzed using ImageJ software. The area of fibrotic lesions was expressed as a percentage of fibrotic area relative to the whole area.

### 2.5. Real-time reverse transcription polymerase chain reaction (RT-PCR)

Total RNA was purified from the kidney or the cultured HK-2 cell with TRIzol reagent (Invitrogen, Carlsbad, CA) and reverse transcribed into cDNA using Primescript RT master mix (TaKaRa). The relative mRNA levels were detected with quantitative real-time PCR using the SYBR Green PCR Kit (TaKaRa) on ABI PRISM 7300Sequence Detector according to the manufacturer’s instructions. The primers were as follows: GAPDH, forward:5′-CAGTGCCAGCCTCGTCTA T-3′, reverse:5′-AGGGGCCATCCACAGTCTTC-3′; KIM-1, forward:5′-AACGCAGCGATTGTGCATCC-3′, reverse:5′-GTCCATTCACCATGGTAACC-3′;pro-caspase-1, forward:5′-GAGCTGATGTTGACC TCAGAG-3′, reverse:5′-CTGTCAGAAGTCTTGTGCTCTG-3′; IL-1β, forward: 5′-GTGATGTTCCCATTAGACAGC-3′, reverse:5′-CTTTCATCACACAGGACAGG-3′; α-SMA, forward:5′-TGTACCCAGGCATTGCTGACA-3′, reverse:5′-TCTGCTGGAAGGTAGATAAG-3′; E-cadherin, forward:5′-GTCAAACGGCATCTAAAGC-3′, reverse: 5′-CAAAGACCTCCTGGATAAACT-3′; Collagen IV, forward: 5′-CCATCTGTGGACCATGGCTT-3′, reverse:5′-GCGAAGTTGCAGACGTTGTT −3′; Fibronectin, Forward: 5′-CTTGCGGGCACCAGACCT-3′, Reverse:5′-CTTCATCCGAGTGTCTGTCT-3′. The GAPDH was the internal control gene. The relative quantity of mRNA expression was calculated by the 2^−ΔΔCT^method and normalized to controls.

### 2.6. Immunohistochemistry

The 4-μm thick paraffin sections of kidneys were deparaffinized in xylene and hydrated in graded ethanol and treated with 3% H_2_O_2_ for 10 min to inhibit endogenous peroxidase activity. Then the sections were blocked with 10% normal goat serum for 40 min. The slides were incubated with primary anti-NLRP3 (1:100), anti-ASC (1:100), anti- Caspase-1 (1:100) and IL-1β (1:100) at 4 °C overnight. On the second day, horseradish peroxides (HRP)-labeled goat anti-rabbit/mouse secondary antibody were added to the sections at 37 °C for 60 min. Finally, immunostaining was visualized using 3, 3-diaminobenzidine (DAB) and hematoxylin counterstaining. Image Pro-Plus Software (Media Cybernetics, Rockville, MD) was used to analyze and calculate their expression.

### 2.7. Western blotting analysis

Proteins extracted from kidney or cultured HK-2 cells were separated by 10–12% SDS-PAGE, transferred onto PVDF membrane, and blocked with 5% nonfat milk. The PVDF membranes were incubated with 1^st^ antibodies of KIM (1:500, Abcam, USA), collagen I (1:1000, Santa Cruz Biotechnology), collagen IV (1:1000, Santa Cruz Biotechnology), FN (1:1000, Santa Cruz Biotechnology), α-SMA (1:1000, Santa Cruz Biotechnology), E-cadherin (1:1000, Cell Signaling Technology), NLRP3 (1:1000, Santa Cruz Biotechnology), ASC (1:1000, Santa Cruz Biotechnology), Caspase-1 (1:1000, Cell Signaling Technology and IL-1β (1:1000, Cell Signaling Technology) overnight at 4 °C. After washing three times with TBST, the PVDF membranes were incubated with 2^nd^ antibodies at room temperature for 1 h, and then the protein bands were observed by enhanced chemiluminescence (ECL) (Thermo, Rockford, USA). Densitometric analysis was performed using the Bio-Rad Quantity One Software. The relative expression of each target protein was normalized to the GAPDH band.

### 2.8. Immunofluorescence

Frozen kidney sections (6 μm) were fixed in 100% acetone for 5 min and blocked with 5% normal bovine serum at room temperature for 60 min. Then the sections were incubated overnight with 1^st^ antibodies (rabbit anti-E-cadherin 1:200, mouse anti-α-SMA 1:200) at 4 °C. After being washed with PBS three times, the sections were incubated with 2^nd^ antibodies (Alexa Fluor 488 goat anti-rabbit 1-1:200; Alexa Fluor 549 anti-mouse 1:200) and counterstained with DAPI. Fluorescence microscopy was used to analyze 10 fields of vision in each kidney.

### 2.9. Statistical analysis

The results of all continuous variables were expressed as mean ± standard deviation (SD). Statistical analysis was performed using ANOVA to compare differences among three and more groups and unpaired Student's *t*-tests to compare differences between two groups using GraphPad Prism 6.0 software (GraphPad Software Inc., San Diego, CA, USA). *p* < 0.05 was defined as statistically significant.

## 3. Results

### 3.1. Effects of berberine on metabolic parameters

As shown in Table 1, the levels of blood glucose, HbA_1_C, TC and TG in the DM group were significantly higher than NC group, (*p* < 0.05), but there was no significant difference between the DM group and BBR group (*p* > 0.05). The ratio of KW/BW and ACR in DM rats was significantly higher than those in the NC group but markedly decreased after BBR treatment (*p* < 0.01). Serum creatinine and BUN were no significant differences among these three groups (*p* > 0.05).

**Table 1.** Metabolic parameters of the rats in different groups (Mean ± SD).

	NC (*n* = 10)	DM (*n* = 10)	DM + BBR (*n* = 10)
Blood glucose (mmol/L)	6.1 ± 0.6	25.9 ± 2.1	25.3 ± 3.1
TC (mmol/L)	3.2 ± 1.4	6.3 ± 2.0*	6.1 ± 2.3*,^#^
TG (mmol/L)	1.4 ± 0.9	4.2 ± 1.2*	4.2 ± 1.3*,^#^
KW/BW	3.13 ± 0.31	6.89 ± 0.36*	4.23 ± 0.29*,^#^
24h urine protein	38.6 ± 8.9	235.8 ± 18.1*	132.6 ± 17.6*,^#^
NAG	10.4 ± 4.8	59.6 ± 7.4*	31.1 ± 6.3*,^#^
BUN (mmol/L)	5.9 ± 2.6	5.8 ± 2.1	5.7 ± 1.9
Scr (mmol/L)	49.3 ± 5.2	47.1 ± 5.3	48.7 ± 7.1

Abbreviations: TC, total cholesterol; TG, total triglycerides; BUN, blood urea nitrogen; Scr, serum creatinine; NAG, N-acetyl-glucosaminidase; NC, normal control group; DM, diabetic group; DM + BBR, Berberine-treated diabetic group. *p < 0.05 between the NC and the DM groups; #*p* < 0.05 between the DM and the DM + BBR groups.

### 3.2. Berberine attenuated renal tubulointerstitial fibrosis in diabetic nephropathy

**Figure 1.**
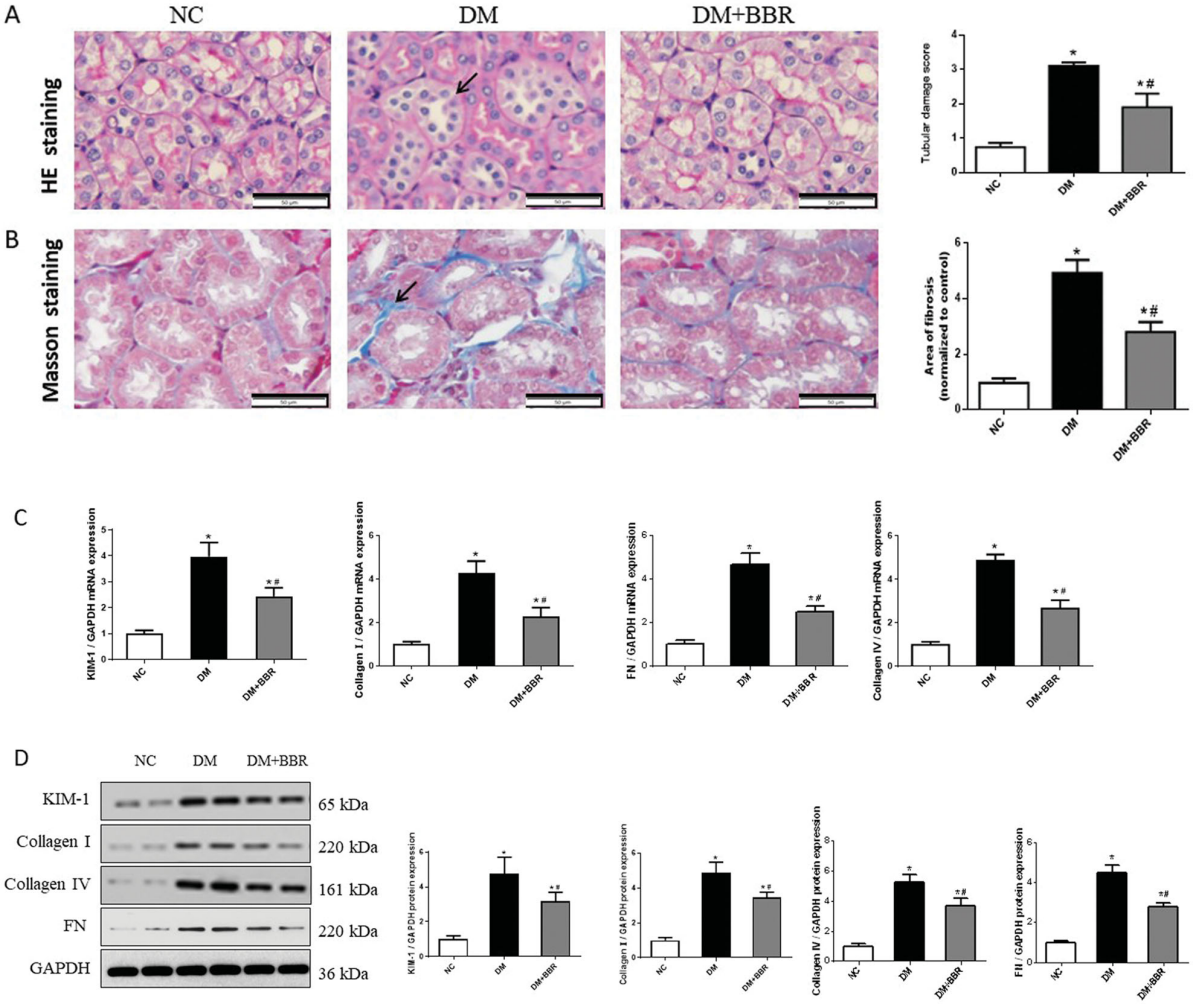
Effects of berberine on renal tubulointerstitial fibrosis. (A) Hematoxylin eosin (HE) staining. (B) Masson’s staining. (C) The mRNA expression levels of KIM-1, Collagen I, Collagen IV and Fibronectin. (D) Western blotting of KIM-1, Collagen I, Collagen IV and Fibronectin proteins (FN) in different groups. NC, normal control group; DM, diabetic group; DM + BBR, berberine-treated diabetic group. *P < 0.05 between the NC and the DM groups; ^#^*P* < 0.05 between the DM and the DM + BBR groups.

HE staining showed that BBR treatment significantly reduced tubular atrophy and inflammatory cell infiltration compared with the DM rats (Figure 1A). Masson’s trichrome staining revealed that compared with the NC group, collagen deposition in tubulointerstitium of diabetic rats increased (*p* < 0.05), while BBR treatment significantly reduced the collagen deposition (Figure 1B, *p* < 0.05). Consistent with this result, western blot analysis and RT-PCR showed that the protein and mRNA levels of Kim-1, collagen I, collagen IV and FN in the kidney were significantly increased in the DM group, but partially decreased after BBR treatment (Figure 1C,D, *p* < 0.05).

### 3.3. Effect of berberine on EMT in diabetic nephropathy

**Figure 2.**
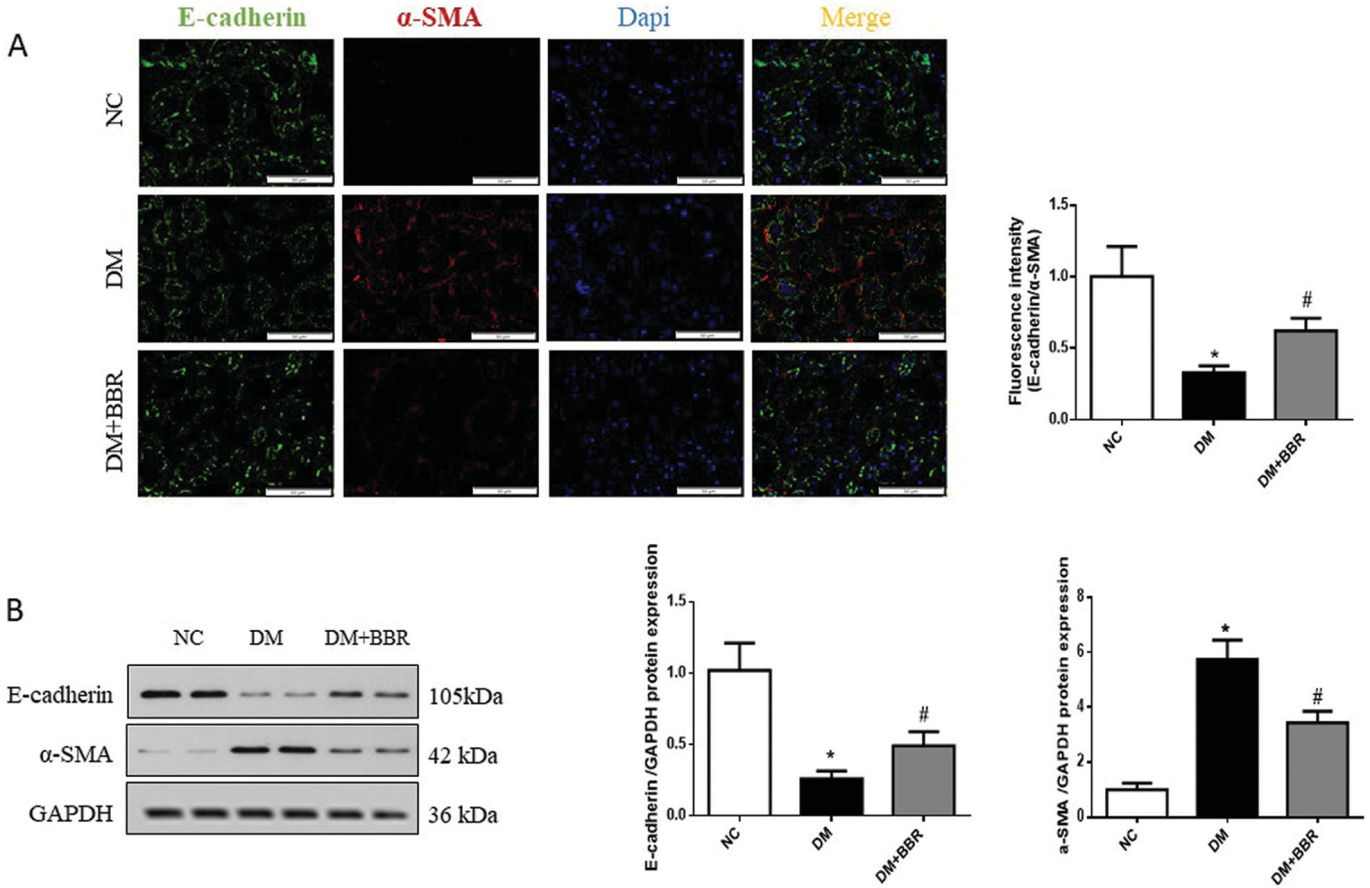
Effect of berberine on epithelial-to-mesenchymal transition. (A) Immunofluorescence for E-cadherin and alpha-smooth muscle actin (a-SMA). (B) Western blotting of E-cadherin and α-SMA proteins in different groups. NC, normal control group; DM, diabetic group; DM + BBR, berberine-treated diabetic group. *P < 0.05 between the NC and the DM groups; ^#^*P* < 0.05 between the DM and the DM + BBR groups.

In the present study, the expression levels of E-cadherin and α-SMA in the kidney were examined by immunofluorescence and western blot (WB). Immunofluorescence results demonstrated that the expression of E-cadherin and a-SMA in the DM group were significantly decreased and increased, respectively, however, BBR treatment partially reversed these changes (Figure 2A, *p* < 0.05). The protein level of E-cadherin was decreased in the kidney of the DM group compared with the NC group, and it was partially recovered by BBR treatment, in addition, the expression of a-SMA was significantly increased in the DM group compared with the NC group, however, BBR treatment significantly decreased the expression of a-SMA (Figure 2B, *p* < 0.05).

### 3.4. Effects of berberine on NLRP3 inflammasome activation in the kidney of diabetic rats

**Figure 3.**
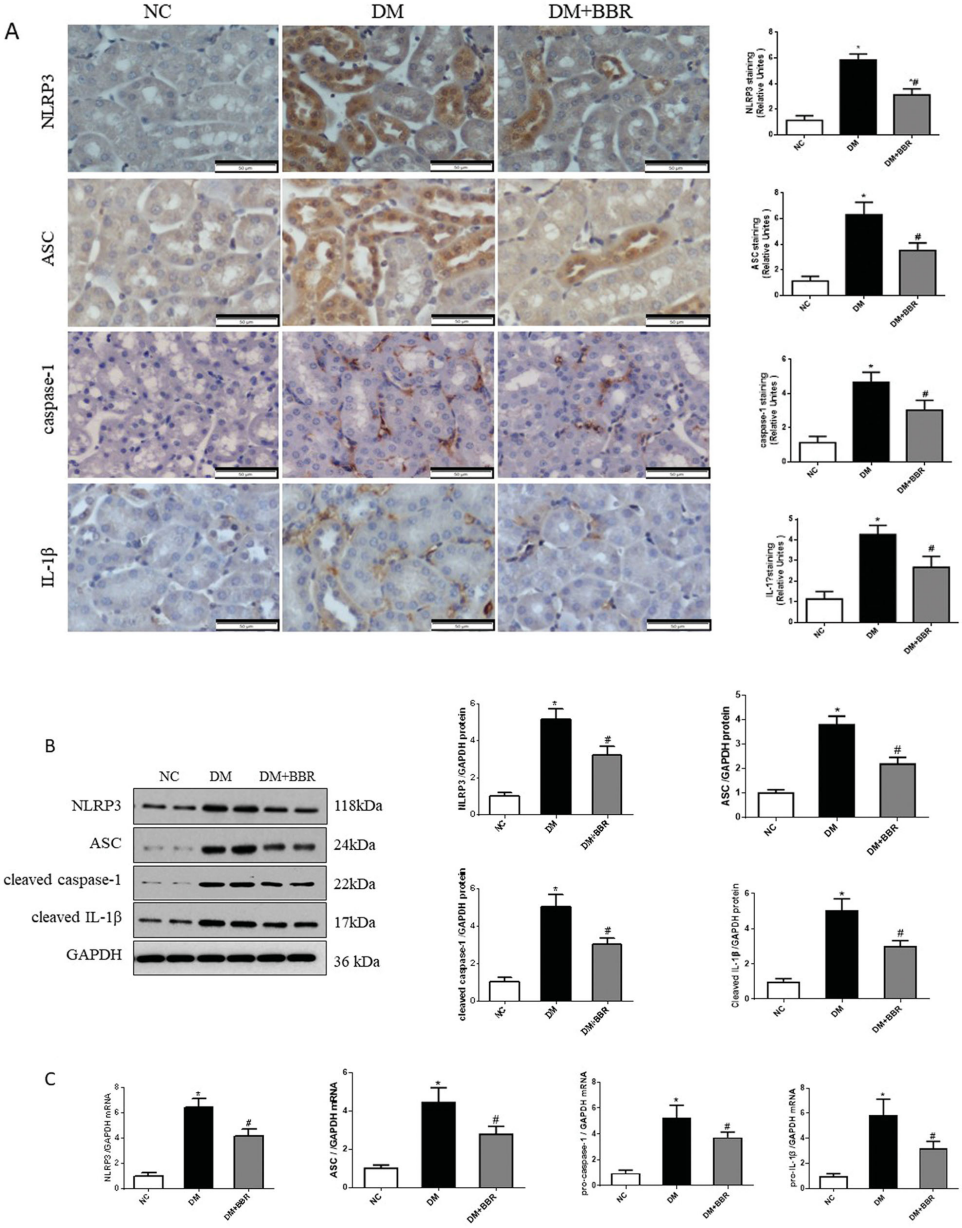
Effect of berberine on NLRP3 inflammasome in the kidney. (A) Immunostaining for the nucleotide-binding oligomerization domain-like receptor protein 3 (NLRP3), apoptosis-associated speck-like protein containing a CARD (ASC), Caspase-1 and interleukin-1beta (IL-1β) protein. (B) Western blot of NLRP3, ASC, Caspase-1 and IL-1β proteins in different groups. (C) The mRNA expression levels of NLRP3, ASC, Caspase-1 and IL-1β. NC, normal control group; DM, diabetic group; DM + BBR, berberine-treated diabetic group. *P < 0.05 between the NC and the DM groups; ^#^*P* < 0.05 between the DM and the DM + BBR groups.

Western blot analysis showed that compared with the NC group, the protein levels of NLRP3, ASC, Cleaved caspase-1 and Cleaved IL-1β were significantly increased in the DM group, while BBR treatment reduced the expression of NLRP3, ASC, Cleaved caspase-1 and Cleaved IL-1β (Figure 3B, *p* < 0.05). Furthermore, we examined the mRNA levels of NLRP3, ASC, pro-caspase-1 and pro-IL-1β in the kidney by real-time PCR. The mRNA levels of NLRP3, ASC, pro-caspase-1 and pro-IL-1β were all significantly upregulated in the DM group compared with the NC group, while those in the BBR treatment group were down-regulated (Figure 3C, *p* < 0.05). The above results indicated that BBR treatment could suppress the activation of NLRP3 inflammasome in diabetic rats.

### 3.5. Berberine reversed high glucose-induced EMT in HK-2 cell

**Figure 4.**
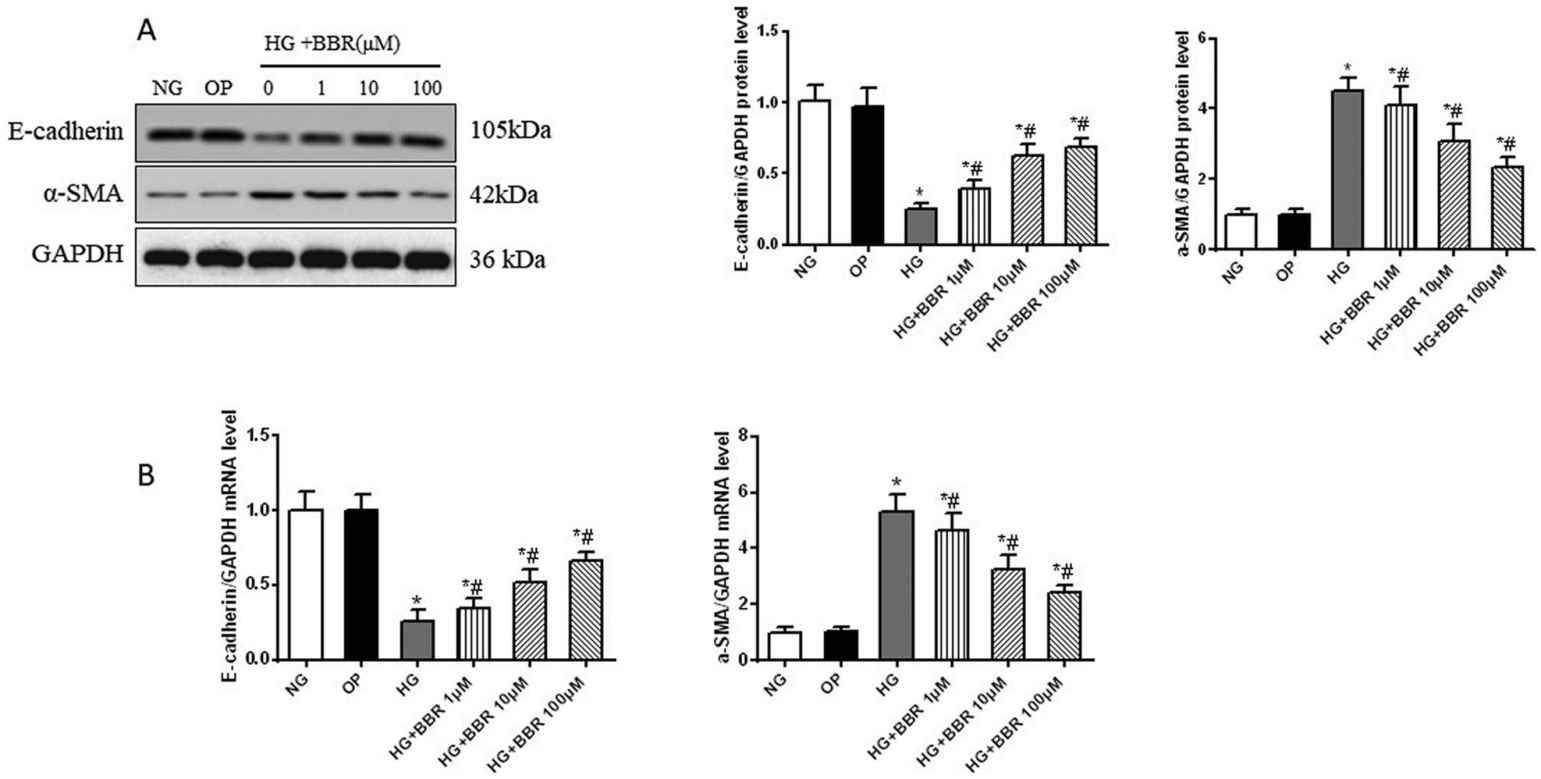
Effect of berberine on epithelial-to-mesenchymal transition induced by high glucose in HK2. (A) Western blot of E-cadherin and alpha-smooth muscle actin (a-SMA) protein induced by high glucose in human renal proximal tubular epithelial cell (HK-2). (B) The mRNA levels of E-cadherin and α-SMA induced by high glucose in HK2. NG, normal control group; OP, osmotic pressure control group; HG, high glucose group; HG + BBR 1μM, high glucose with berberine 1μM treatment group; HG + BBR 10μM group, high glucose with berberine10μM treatment group; HG + BBR 100μM, high glucose with berberine100μM treatment group. *P < 0.05 vs. NG group, ^#^*P* < 0.05 vs. HG group.

HK-2 cells were incubated with high glucose for 48 h, and were harvested for protein and mRNA analysis. As shown in Figure 4A,B, high glucose could decrease the protein and mRNA levels of E-cadherin and increase the expression of α-SMA (*p* < 0.05), while BBR could reverse these changes in a dose-dependent manner (*p* < 0.05). These data suggested that BBR could inhibit HG-induced EMT in HK-2 cells.

### 3.6. Effects of berberine on the expression of NLRP3 inflammasome in HK-2 cells

**Figure 5.**
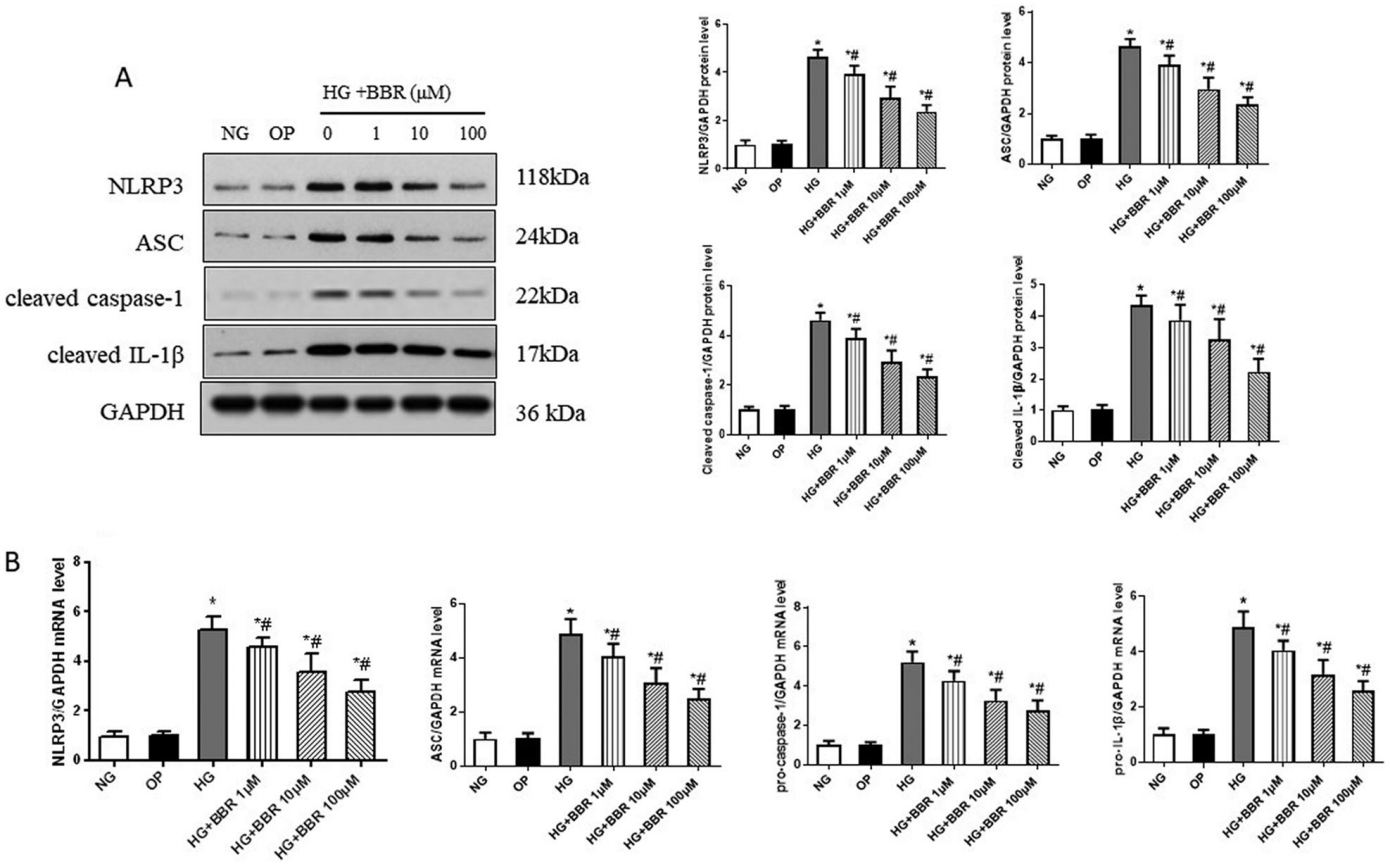
Effect of berberine on NLRP3 inflammasome induced by high glucose in HK2. (A) Western blot of the nucleotide-binding oligomerization domain-like receptor protein 3 (NLRP3), apoptosis-associated speck-like protein containing a CARD (ASC), Caspase-1 and interleukin-1beta (IL-1β) protein induced by high glucose in human renal proximal tubular epithelial cell (HK-2). (B) The mRNA levels of NLRP3, ASC, Caspase-1 and IL-1β are induced by high glucose in HK2. NG, normal control group; OP, osmotic pressure control group; HG, high glucose group; HG + BBR 1μM, high glucose with berberine 1μM treatment group; HG + BBR 10μM group, high glucose with berberine10μM treatment group; HG + BBR 100μM, high glucose with berberine100μM treatment group. *P < 0.05 vs. NG group, ^#^*P* < 0.05 vs. HG group.

**Figure 6.**
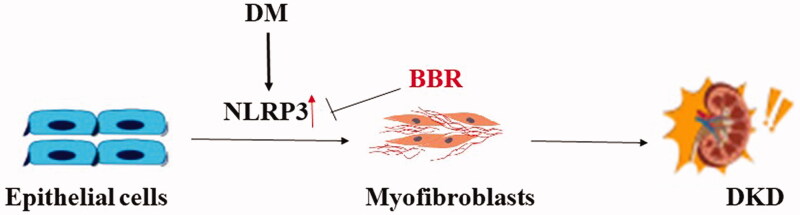
A schematic model of the pathway showing the inhibitory effect of BBR on the renal inflammatory and fibrotic processes.

To determine the effects of berberine on the expression of NLRP3 inflammasome in HG-induced HK-2 cells, the expression of protein and mRNA of NLRP3 inflammasome was measured using western blotting and RT-PCR. Both protein and mRNA levels of NLRP3, ASC, Caspase-1 and IL-1β were all markedly higher in the HG-induced group compared with the NG group; however, treatment with BBR reduced the expression of NLRP3, ASC, Caspase-1 and IL-1β in a dose-dependent manner (Figure 5A,B, *p* < 0.05). These results of the cell experiment were consistent with the results of the animal experiment, which indicated that BBR could inhibit HG-induced EMT involving the inactivation of the NLRP3 inflammasome (Figure 6).

## 4. Discussion

DKD is believed to be one of the most serious microvascular complications of diabetes, which is highly prevalent in ESRD. At present, western medicine mainly focuses on controlling blood glucose, hypertension and inhibiting the renin-angiotensin system, which can only partially ameliorate but not prevent the development of renal failure [23]. BBR is extracted from Chinese herbs, which has a potential clinical application as a drug for the treatment of diabetes and its complications. In the present study, we demonstrated that BBR could ameliorate renal tubulointerstitial fibrosis by inhibiting hyperglycemia-induced EMT *via* inactivation of NLRP3 inflammasome both *in vivo* and *in vitro*.

Although the glomerulus, especially mesangium, has always been the focus of DKD, tubulointerstitial injury is also a major feature of DKD and an important predictor of renal dysfunction [24–26]. This pathological phenomenon occurs due to the accumulation of interstitial ECM such as fibronectin, type I collagen and type IV collagen in the kidney. Although the origin of myofibroblasts is still controversial, EMT is considered as the main source of myofibroblasts. In DKD, EMT of renal tubular epithelial cells is believed to participate in the progress of tubulointerstitial fibrosis through the accumulation of renal matrix protein. EMT induced by hyperglycemia is generally considered to be the initial factor leading to matrix accumulation and deposition, so EMT is also considered to be an important mechanism of renal fibrosis and renal dysfunction in DKD [27]. Therefore, targeting EMT is considered a potential therapy to delay the progression of tubulointerstitial fibrosis in DKD [28].

The current study demonstrated that the expression of α-SMA was significantly decreased and the expression of E-cadherin was increased in BBR-treated diabetic rats, suggesting that the inhibition of EMT might be an important mechanism for BBR to exert a renoprotective effect and inhibit the progression of tubulointerstitial injury.

The activation of NLRP3 inflammasome plays an important role in initiating and aggravating diabetic tubulointerstitial injury [29]. The NLRP3 inflammasome complex activates caspase-1 and then increases the production of some inflammatory cytokines, such as IL-1β, MCP-1 and VCAM-1. Furthermore, the inflammatory cytokines IL-1β, MCP-1 and VCAM-1 play an important role in mediating the tubulointerstitial injury [30]. It is becoming more evident that targeting the NLRP3 inflammasome is considered a potential therapeutic approach for DKD [31,32].

Previous studies have shown that BBR has a wide range of pharmacological activities and has been demonstrated to exert protective effects against DN progression by ameliorating a variety of pathological changes. Such as, BBR could reduce renal injury and inflammatory response, and podocyte apoptosis by inhibiting TLR4/NF-κB pathway [33]. Additionally, BBR could protect glomerular podocytes *via* positively regulating Drp1-mediated mitochondrial dynamics [20]. In this study, we observed that diabetic rats displayed significant renal tubulointerstitial injury and fibrosis, meanwhile, the NLRP3 inflammasome was activated as indicated by the increased expression of NLRP3, ASC, Caspase-1 and IL-1β. While berberine obviously suppressed the activation of the NLRP3 inflammasome by decreasing the expression of NLRP3, ASC, caspase-1 and IL-1β in diabetic rats. Our vitro experiments also indicated that berberine could suppress the activation of NLRP3 inflammasome. These findings support that administration of berberine may be a potential therapeutic for DKD by inhibiting NLRP3 inflammasome. Our study further revealed that BBR could inhibit high glucose-induced tubular epithelial-to-mesenchymal transition and renal interstitial fibrosis by suppressing NLRP3 inflammasome.

In conclusion, our study demonstrates that berberine can effectively inhibit EMT and renal interstitial fibrosis induced by high glucose by blocking NLRP3 inflammation. These findings suggest that berberine can be used as a new therapeutic drug to ameliorate tubulointerstitial fibrosis in DKD.
